# Health Tract, No. 67

**Published:** 1865

**Authors:** 


### HEALTH TRACT, No. 67.

### PAIN

Is a blessing, being Nature's admonition that something is wrong, and impels to its rectification. If, for example, there were no feeling in the fingers or feet, they might any night be frozen or burnt off, and we would wake in the morning to a life-long deformity.

The immediate cause of all pain is in the condition of the blood acting on the nerves, it being too thick, too abundant, or too poor. If too poor ,it must be enriched by the introduction of iron into the system. When too abundant, it must be lessened in quantity by working it off in exercise, and by diminishing its supply, which is furnished by the food eaten. When too thick. which is the same as being impure, it must be remedied by a large and daily exposure to the fresh, pure, out-door air, because every breath goes in pure, and as it were empty, but comes out loaded with impurity. Hence animals, being out of doors all the time, remain still, and without exercise get well, because, breathing a pure air, every breath is directly remedial.

The more fixed and severe a pain is, the more dangerous it is, as it will soon cause destruction of the parts. When pain is shifting, it is only functional, and arises merely from a surplus of blood in the veins or arteries, pressing against the nerves of the part. In some cases, accumulations of wind or gases cause pain. Pain being the result of too much blood in a part, as a very general rule, the remedy, in severe and pressing cases, is to apply a mustard-plaster near that part, which draws the blood away, as is seen by the reddening of the skin.

The most agonizing pains are often removed in the twinkling of an eye, by dipping a bit of cloth (woolen, flannel, or cotton) in a mixture of equal parts of sweet oil, chloroform, and strong spirits of hartshorn just shaken together, and spread over the spot, with a handkerchief wadded in the hand, and held over the cloth, so as to retain the more volatile ingredients; to be removed the moment the pain ceases.

The safest and most comfortable application in nature for the relief of all pain, especially that arising from inflammation, is a woolen cloth kept, very warm, even hot, by the steady addition of hot water, or a stream of warm water, where the painful part admits it. When pain is severe, sharp, or thrilling, there is inflammation, and arises from there being too much blood in the arteries; if dull and heavy, it is caused from there being too much blood in the veins.

The pain of inflammation gives heat; hence, headache with a hot head, is from too much blood in the arteries, and there is throbbing; draw it away by putting the feet in very hot water; this often removes pain in any part of the body above the ankles.

When there is too much blood in the veins of the head, there is a dull pain or great depression of spirits, and the feet are always cold. It is this excess of blood in the veins of the head or brain, which always induces the despondency which so frequently causes suicide. When this is attempted by cutting the throat, the relief is instantaneous, and the victim becomes anxious for the life he had just attempted to destroy. Hence, a good out-door walk, or a hot bath, a sudden fit of laughter, or a terrible burst of passion, by dispersing the blood to the surface from the centers, puts the Blues and Megrims to flight also.

